# Cultural Competence Interventions in European Healthcare: A Scoping Review

**DOI:** 10.3390/healthcare12101040

**Published:** 2024-05-17

**Authors:** Berta De-María, Gabriela Topa, M. Angeles López-González

**Affiliations:** 1 Department of Social and Organizational Psychology, Faculty of Psychology, Universidad Nacional de Educación a Distancia (UNED), 28040 Madrid, Spain; bmm@psi.uned.es (B.D.-M.); gtopa@psi.uned.es (G.T.); 2 Psychology Department, Faculty of Health Sciences, Universidad Rey Juan Carlos, 28922 Madrid, Spain

**Keywords:** cultural competence, healthcare, interventions, Europe, scoping review

## Abstract

Europe is undergoing rapid social change and is distinguished by its cultural superdiversity. Healthcare is facing an increasing need for professionals to adapt to this environment. Thus, the promotion of cultural competence in healthcare has become a priority. However, the training being developed and their suitability for the European context are not well known. The aim of this qualitative study has been to map the scientific literature in order to comprehend the current state of research on this topic. For this purpose, we conducted a systematic scoping review of the empirical publications focused on cultural competence interventions for healthcare professionals in European countries. The search was conducted in eight thematic (PsycINFO, MedLine, and PubPsych) and multidisciplinary databases (Academic Search Ultimate, E-Journals, Scopus, ProQuest, and Web of Science) to identify relevant publications up to 2023. Results were presented qualitatively. Out of the initial 6506 records screened, a total of 63 publications were included. Although the interventions were implemented in 23 different European countries, cultural competence interventions have not been widely adopted in Europe. Significant heterogeneity was observed in the conception and operacionalización of cultural competence models and in the implementation of the interventions. The interventions have mostly aimed at improving healthcare for minority population groups and have focused on the racial and ethnic dimensions of the individual. Future research is needed to contribute to the conceptual development of cultural competence to design programs tailored to European superdiversity. This scoping review has been registered in OSF and is available for consultation.

## 1. Introduction

We find ourselves in an era of unprecedented global mobility, where the number of people residing in a country different from their country of origin has reached historical levels. In fact, the International Organization for Migration estimates that approximately 281 million migrants moved internationally during the year 2022 [1]. For instance, Europe hosts people from over a hundred different nationalities, with more than 20 recognized official languages and over 140 languages in use [2]. We can affirm that societies are culturally diverse and face considerable challenges stemming from growing issues of inequality and social discrimination [3].

In the healthcare domain, inequities represent a serious social problem with negative consequences, especially for those who experience them. These repercussions range from reduced utilization of healthcare services [4,5] to barriers in accessing disease prevention and treatment programs [6], poor health outcomes [7], and a lower perception of the quality of care [8]. Furthermore, at organizational, structural, and clinical levels, there are also observed losses of resources and opportunities [9].

In response to the growing cultural diversity, healthcare systems are paying increased attention to the need for providing services tailored to plurality. In this context, the culture and cultural identity of individuals play a crucial role in healthcare [10]. Consequently, numerous efforts have been made in recent years to reduce social inequalities by establishing culturally competent healthcare systems [11]. This approach poses a challenge, given that healthcare professionals may struggle to understand the personal and social beliefs and meanings that patients from diverse cultures hold about their illness, future expectations, and goals [12]. For example, interpretations of what constitutes good or poor communication may vary among cultures. Numerous studies have identified key factors that can predict patient preferences regarding information and their involvement in the medical process, such as age, gender, education, religion, culture, attire, etc. A study conducted in Australia [13] showed that individuals who believe in divine influence in the illness process tend to prefer less information about their condition. Another study demonstrated how family plays a central role in Egypt and it is considered inappropriate for the physician to disclose the exact diagnosis to the patient; instead, it is acceptable for this information to be relayed to family members. Thus, in some Asian cultures, patients may exhibit attitudes of denial and passivity towards illness, which can lead to decreased treatment adherence [14]. Additionally, in many Asian regions, self-medication is common due to a strong tradition of self-care. Beliefs in reincarnation and miracles can influence how Asian and Arab families cope with illness and death. Understanding the cause of illness varies among cultures: in some Asian cultures, it is associated with bodily imbalance, while in Egyptian society, it is viewed as divine punishment for sin [15]. Consequently, when these explanatory models are not understood, the quality of healthcare may be negatively impacted [16].

To achieve patient understanding and improve healthcare delivery, two complementary strategies have emerged: Patient-Centered Care (PCC) and cultural competence (CC). Both are key to overcoming the deficiencies of the traditional medical model, characterized by its rigidity and insensitivity to individual and cultural differences. However, each originates from different theoretical positions and presents distinct practical implications. PCC places the individual at the center of medical care; it adjusts to the individual needs and desires, incorporating the personal preferences, values, and circumstances of each patient [17]. PCC promotes autonomy and active participation of the individual in their own care [18] and it has been demonstrated that this approach can increase patient satisfaction, empowerment, and engagement, see [19]. However, PCC may vary in practice due to the diversity in conceptual interpretations and existing models. More than a singular approach, PCC seeks to correct the limitations of the traditional medical model, which often disregards patient preferences and needs, and can lead to impersonal treatment [20].

Cultural competence (CC) is generally defined as “an integrated set of knowledge, skills, behaviors, attitudes, practices and congruent policies that converge within a system, organism or among professionals and enable that system, agency or those professions to work effectively in cross-cultural situations” (p. 13, [21]). Cultural competence (CC) entails healthcare professionals recognizing and respecting patients’ cultural differences and adapting their care accordingly. This occurs through a personal development process, where awareness and the acquisition of cultural knowledge lead to the refinement of professional skills [22].

The study of CC originated in the United States in the mid-20th century, propelled by political movements advocating for civil rights, respect for cultural diversity, and concern about existing discrimination against various population groups [23]. Subsequently, its study expanded to other societies with similar characteristics, such as Canada and Australia. From the 1960s and 1970s onward, medical anthropology began to influence healthcare, focusing on the diversity of beliefs and practices related to health [24] and recognizing the need to develop skills and knowledge for working in multicultural contexts. In the 1980s and 1990s, with the increase in migration and globalization, the concept of cultural competence became more relevant, especially in those countries with more diversified populations [20]. The literature of this period showed a growing interest in understanding racial and ethnic disparities in healthcare [25].

This led to the creation of cultural competence training programs for healthcare professionals with the purpose of reducing health inequity and effectively improving the quality of healthcare services for migrant populations with limited English proficiency and limited exposure to Western cultural norms. To overcome these challenges, approaches were proposed that included the use of interpreters and “cultural brokers” to improve intercultural communication [26] and to foster an understanding of the history and norms of different minority groups [20]. At the same time, guidelines were developed to assist healthcare professionals in considering the cultural context of patients and conducting cultural assessments. However, it was soon recognized that, although it is important to value and respect the cultural, religious, and ethnic diversity, educational level, attire, etc., present in many countries, it was not practical for healthcare professionals to be familiar with all cultural perspectives [9]. Furthermore, it was observed that grouping patients categorically posed the risk of stereotyping individuals based on their racial or ethnic group membership [9] and drawing inappropriate assumptions about their beliefs and behaviors [27]. Since stereotypes and prejudices are automatically activated and their unconscious nature makes detection and control impossible, treatment and healthcare could be influenced by the professional’s implicit bias and constitute a significant factor in health disparities [28]. Thus, these early categorical approaches, focused on teaching healthcare professionals about characteristics and behaviors associated with specific cultural groups, fell short due to their tendency to overly simplify culture and not consider its dynamic nature [29].

To address these concerns, more balanced intercultural approaches were proposed. They combined basic knowledge of specific cultural groups with the development of attitudes and skills applicable to any cultural context and all individuals, paying attention to intra-group variability and the impact of factors such as acculturation and socioeconomic status. This entailed a shift from the biomedical model toward the biopsychosocial understanding of health, where patients participate in decision making, receive explanations about the illness and its causes, and cooperate in negotiating treatment plans [20]. Concurrently, it was emphasized that healthcare professionals also bring their own cultural perspectives to clinical encounters. For this reason, healthcare professionals were encouraged to reflect on their own cultural backgrounds, including the privilege and power associated with their professional status, to ensure more inclusive and equitable care [20]. This reinforces the idea that cultural competence not only involves knowledge of cultural differences but also awareness of one’s own attitudes and predispositions. The intercultural approach represents a significant step toward improving healthcare in a diverse world, which has been linked to greater treatment adherence [30]; increased patient satisfaction [31]; and improvement in knowledge, attitudes, and skills of health professionals [32]. It strengthens the ability of health systems and their clinicians to deliver appropriate services to diverse populations, thereby improving outcomes and reducing disparities [33]. As healthcare professionals adopt more inclusive and reflective practices, more equitable and effective healthcare is expected for all [34].

Since its inception, the concept of CC has been framed within an approach particularly focused on the diversity of racial and ethnic minorities, characterized as broad communities of citizens, showing territorial ties and stable social organization, and originating from areas that shared historical or colonial bonds [35]. This is due to the common interpretation of the concept of “culture” as synonymous with race and ethnicity [36]. Nevertheless, culture is a dynamic relational process of shared meanings that originates in the interactions among individuals [37]. It provides norms for perceiving, believing, evaluating, communicating, and acting among those who share a language, a historical period, and a geographical location [38]. These shared elements are transmitted from generation to generation with some modifications, so the historical, social, political, and economic context in which culture is framed is a fundamental factor for its proper understanding [39]. Therefore, the study of cultural competence must be developed within a specific geographical context and at a precise historical moment.

Europe is comprised multiple states, presenting a mosaic of political, linguistic, social, and even environmental characteristics that differentiate it from other countries such as the USA, Canada, or Australia, which were founded by European migrants who conquered and settled in the territories of the indigenous peoples inhabiting those regions. In recent years, Europe has undergone changes in its demographic patterns and social configurations, now characterized by “superdiversity”, a dynamic interplay of variables among an increasing number of new, small and scattered, multiple-origin, transnationally connected, socio-economically differentiated, and legally stratified immigrants (p. 1, [40]). Yet, superdiversity opens the door to individual and identity differences, as well as to multidimensional and overlapping social categories [41]. This implies developing a healthcare sensitivity to individual differences and the intersectionality of identity-shaping factors, valuing other dimensions such as gender, age, religion, social class, etc., and the complexity of their interactions [42].

The study of CC has evolved since its inception in the north American context and research has shown increasing interest in this approach. However, in Europe, the scope of studies on cultural competence is unknown, while there is abundant literature on this subject in other countries: CC healthcare systems (e.g., [11]); CC dimensions and outcomes (e.g., [22]); CC healthcare provider educational interventions (e.g., [29,32]); CC improvement in patient outcomes (e.g., [43]); or success in the implementation and evaluation of CC-based interventions (e.g., [29]). The information available to design CC-based interventions comes from other geographical and cultural areas, which may not provide the best response to European needs. Adequate information on European sociocultural characteristics can lead to better health outcomes for patients. Furthermore, it could improve CC-based academic curricula for health science students. Likewise, it could enable the modification of institutional practices that significantly influence healthcare, such as cultural norms and the social and media perception of cultural diversity. Finally, research results in the European context could lead to policy changes by the governments of these countries, defining priority areas for healthcare at preventive, care, and research levels.

All these aspects raise questions about the current state and evolution of research in Europe. To address these inquiries, we propose conducting a scoping review to analyze scientific publications focused on CC interventions targeting European healthcare professionals. A scoping review, unlike a classic systematic review, focuses on systematically identifying and mapping the breadth of evidence available on a topic. These reviews do not require addressing specific hypotheses or research questions and their main objective is to explore a broad field, clarify concepts, identify key characteristics, and discover areas that require further investigation [44]. Pollock et al. [45] emphasize that scoping reviews are useful for identifying types of evidence, examining methodologies, discovering knowledge gaps, or even serving as a preliminary step to a systematic review. Being more exploratory, scoping reviews allow for a flexible approach, without the obligation to conduct exhaustive critical evaluations or detailed quality analyses, as in systematic reviews. This type of review is ideal for gaining a general perspective and informing future research, helping to guide policy and academic decisions in diverse fields such as CC Interventions in European Healthcare (CCIEH).

The specific objectives are outlined as follows: (a) to identify the scientific production and geographical distribution of CCIEH; (b) to characterize the theoretical models mentioned in the literature on CCIEH; and (c) to describe the elements and strategies of CCIEH, including (i) study methodology, target population, research design, assessment instruments, and measured variables, and (ii) characteristics of interventions, format, duration, components, and performance tests. The ultimate goal is to provide a comprehensive mapping of CCIEH and identify research gaps to contribute to future lines of research.

## 2. Method

This scoping review has been developed by establishing a protocol based on both the PRISMA-ScR checklist recommendations (see Appendix A) and the JBI Manual for Evidence Synthesis on one hand (i.e., [46,47]) and the prior procedures developed by the research group on the other hand (i.e., [48]). Prior to the review commencement, a protocol was implemented with the primary aim of ensuring methodological quality and rigor. As a first step, the scope of the review was meticulously defined, outlining inclusion and exclusion criteria in detail. Subsequently, a series of exploratory literature searches were conducted before constructing the final search equation. Throughout this process, the research team collaborated dynamically by establishing a shared workspace and holding regular meetings.

These sessions were used as a space to assess the progress of the review, address challenges encountered during data extraction and analysis and ensure consistency in the application of data extraction tools. Simultaneously, active stakeholder participation was encouraged throughout the development of the review. This was performed by aiming to integrate ideas generated by those individuals who provided valuable perspectives and contributed to the relevance and applicability of the results. Additionally, a guide was prepared, which included the essential elements and components for addressing the research question and identifying relevant sections in the primary documents to streamline the process of data extraction and coding of records. The resulting model was initially registered in the collaborative project management tool OSF, dated 17 May 2023, and is available for consultation (https://archive.org/details/osf-registrations-wjh6y-v1) (accessed on 17 May 2023).

### 2.1. Inclusion and Exclusion Criteria

The inclusion and exclusion criteria are based on the research question, implementing an adaptation of the PICO strategy called “dS-CoCIP” (documents, Studies, Concept, Context, Intervention, and Participants) and following the methodological guidelines proposed for systematic reviews in health sciences [48].

Documents: We included (a) periodical (journal articles) and (b) non-periodical publications (books, book chapters, and doctoral theses). Documents that are not considered traditional academic sources were excluded, such as editorials, abstracts, conference presentations, indexes, videos, podcasts, posters, and other popular publications.

Studies: Studies based on empirical research were selected, where conclusions were strictly drawn from concrete and verifiable evidence, both quantitative and qualitative, and where the data were primary and original. Case studies, theoretical essays, and narrative and/or systematic reviews were discarded.

Concept: The phenomenon of interest was cultural competence (CC), defined as “a set of knowledge, skills, behaviors, attitudes, practices and congruent policies that converge within a system, organism or among professionals and enable that system, agency or those professions to work effectively in cross-cultural situations” (p. 13, [21]).

Context: Specific consideration has been given to publications focused on healthcare, with a geographic limitation to the European region.

Intervention: The review will include interventions conducted with the aim of enhancing CC. These interventions may address aspects such as fostering cultural sensitivity, understanding diversity, and developing intercultural communication skills. It is required that interventions are clearly detailed and evaluated, specifying the strategies employed. Implementation formats will not be predetermined or limited in advance.

Participants: They include both practicing professionals and students undergoing training in the healthcare sector. These groups are responsible for providing preventive, curative, therapeutic, and/or rehabilitative care to patients. They are experts with specialized training in various areas such as medicine, nursing, pharmacy, clinical psychology, physical and occupational therapy, social work, and medical technology, among others. Additionally, it includes other workers who provide direct personal care services in healthcare and residential settings [49].

### 2.2. Search Strategy

The authors of the research (BMM, MALG, and GTC) established a strategy after a preliminary phase of exploratory searches. This phase was crucial for identifying relevant studies and for analyzing the dynamics of keywords that could generate noise or false positives. Subsequently, following procedures used in previous studies (e.g., [50,51]), Boolean exclusion operators, such as NOT, were applied to refine the search and systematically eliminate works that did not meet the criteria of interest. This ensured an accurate, complete, and efficient selection that effectively aligned with the research objectives.

The final search equation was constructed using Boolean operators (AND, OR, and NOT) and truncation (* and quotation marks) across eight automated databases, as can be seen in the Supplementary Table S1.

### 2.3. Sources of Information

The bibliographic search was conducted in April 2024 and the search period encompassed all records available up to December 2023. No language restrictions were established. Therefore, records found in languages other than English were translated using the online tool www.deepl.com.

The documents were retrieved using formal, informal, and retrospective search strategies.

Formal strategies: (a) Thematic databases: thematic healthcare content databases were consulted: PsycINFO, MedLine, and PubPsych; (b) multidisciplinary databases, specifically: Academic Search Ultimate, E-Journals, Scopus, ProQuest, and Web of Science; and (c) review of the bibliographic references of the retrieved articles.

Informal strategies: Manual searches were conducted on various academic social networks, including Google Scholar, ResearchGate, and Academia.edu, with the aim of analyzing and collecting relevant records. 

Retrospective strategies: Previous systematic reviews and meta-analyses were explored to locate potentially relevant records.

### 2.4. Data Extraction and Codification

The records obtained from each database were exported to a bibliographic reference manager (EndNote 20) as separate files. Subsequently, a library named “SR_CC” was created where all records were grouped and duplicates resulting from the use of multiple databases were removed. Afterward, the metadata was exported to an Excel spreadsheet format for further analysis. Initial meetings were held among the three researchers to define the approach of the work and to agree upon a preliminary protocol, called “dS-CoCIP”, which established the fields of analysis, and the order and criteria for the inclusion and exclusion of records. Simultaneously, an internal guide was developed to address and share difficulties as they arose and to refine the protocol. The first 10 records were jointly analyzed by the team to identify possible issues and establish a unified workflow process. Subsequently, two team members (BMM and MALG) independently analyzed the remaining records in parallel. All records were qualitatively evaluated through a complete document review. Abstracts were only consulted when their content justified the exclusion of a record and its reasons. Regular meetings were scheduled to discuss the results of the analysis. To encourage collaboration, a WhatsApp group was created where encountered difficulties were shared and significant findings requiring follow-up were discussed. The final database contained bibliometric metadata including the (a) record code, (b) authorship, (c) year of publication, (d) document title, (e) journal or book name, (f) DOI, and (g) abstract. Additionally, additional fields were added to complete the “dS-CoCIP” protocol: (a) document typology (d), which distinguished between journal articles, books, book chapters, and doctoral theses; (b) study type (S), with a classification including empirical studies, theoretical studies, reviews, and case studies; (c) concept (Co), to determine if the document was related to a specific construct, specifying the theoretical framework and providing an explicit definition; (d) context (C), which comprised two variables: (i) setting, to indicate the study context and (ii) geographic context, indicating the continent and, in Europe, the nationality of the analyzed sample; (e) intervention (I), detailing the content, structure, duration, methodology, and evaluation of interventions aimed at European healthcare personnel; and (f) participants (P), containing detailed information about the involved professionals, such as discipline and sample size.

### 2.5. Data Synthesis

The data are presented through qualitative narrative synthesis. Thus, a content analysis of the variables included in the study was conducted: (a) exploration of scientific production and geographical distribution of publications, (b) conceptual analysis of the terminology used, and (c) study of the characteristics of experimental interventions. The following procedure was followed to conduct the different analyses: (i) generation of a list of variables measured in the titles, abstracts, and full texts of the documents; (ii) description of the study variables, capturing the main characteristics in each selected publication; and (iii) derivation of general conclusions from the individual data. Descriptive statistical techniques were used to summarize the obtained data.

Finally, the results of the systematic exploration of the works under study have been presented narratively, accompanied by appropriate visual tools for the type of research conducted [43]: diagrams, graphs, tables, and infographics created using Microsoft Excel (version 2402), PowerPoint (version 2402), Canva (version 2.5), and Meta-Chart (version 1.0).

## 3. Results

### 3.1. Scientific Production, Temporal Evolution, and Geographic Distribution

**Figure 1:**
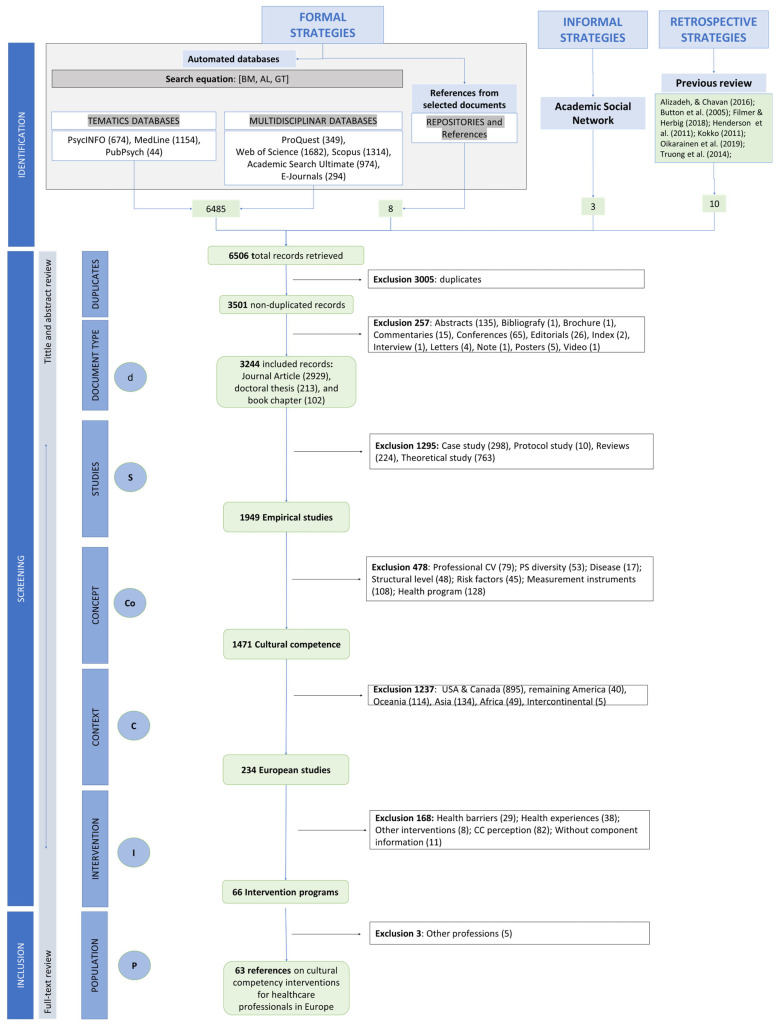
Flowchart of the review process [22,52,53,54,55,56,57].

The search conducted identified 6506 publications: 63 publications met the inclusion criteria, which represents 0.97% of the total. Figure 1 illustrates the complete process undertaken: the selection of formal and informal strategies, the details of each record, and the reasons for inclusion and exclusion in the different phases of the screening process, in accordance with the “dS-CoCIP” study protocol.

Firstly, it is advisable to differentiate between publications and interventions to analyze the results and prevent potential biases in representativeness and distortion of the variables under analysis. Although 63 publications were identified, the total number of CCI for European health professionals was 54. This is due to the fact that six interventions have generated several publications and analyses, developed in different documents. (1) The Netherlands (e.g., [58,59,60,61]); (2) Sweden (e.g., [62,63]); (3) United Kingdom (e.g., [64,65,66,67,68]); and (4) Denmark (e.g., [69,70,71,72]).

**Figure 2:**
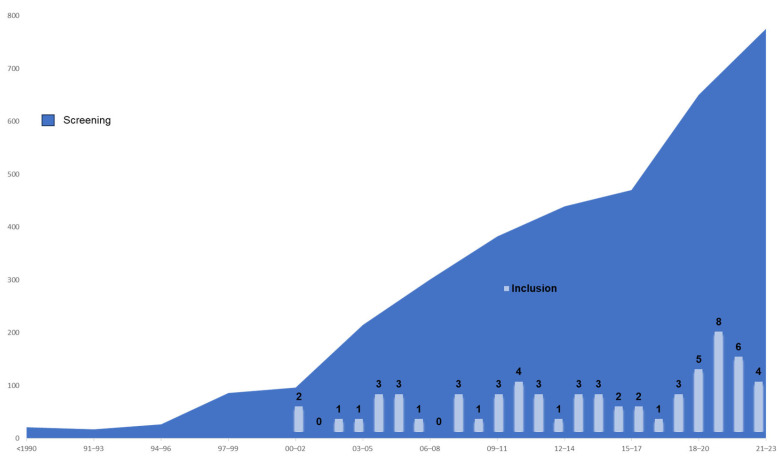
Evolution of publications on cultural competence interventions in European Healthcare.

In Figure 2, the contrast in the temporal evolution between the publications found and those selected in the bibliographic search is illustrated. The first two retrieved publications relate to the year 2000. From that date onward, the time series shows an irregular evolution, with a peak of eight publications in the year 2021. More detailed information about each of the references can be found in Appendix B.

**Figure 3:**
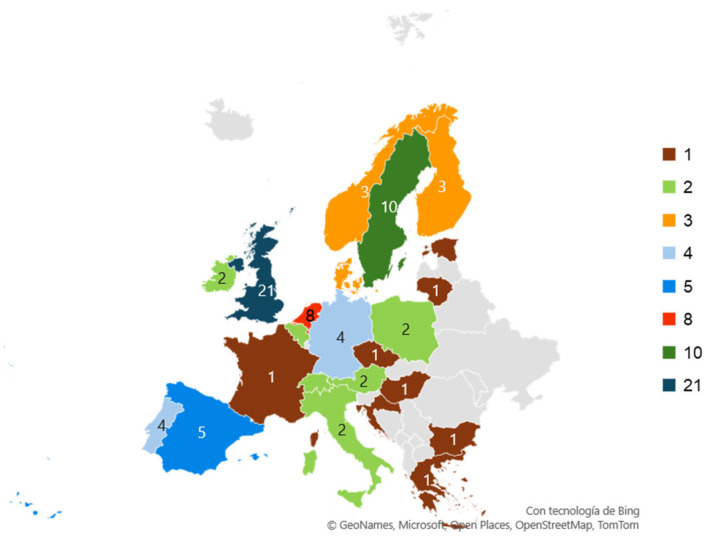
Geographical distribution of cultural competence interventions in European Healthcare *Note*: the unit of measure is CCIEH. It should be noted that the number of publications (63) does not correspond to the total number of interventions, since there have been six multinational CCIEH involving several European countries.

Lastly, concerning the geographical distribution of CCIEH, Figure 3 and Appendix B display the European countries that have participated in both national programs and multinational projects. On the one hand, at an individual level, among the 23 participating countries in CCIEH, 3 stand out for having a higher number of publications: the United Kingdom participated in 39% of CCIEH, Sweden in 18%, and the Netherlands in 15%. On the other hand, six CCIEH have consisted of multinational projects, involving several countries, ranging from 3 to 13. Thus, 57% of the analyzed countries would have participated solely in this type of CCIEH. Regarding the geographical regions into which Europe has traditionally been subdivided, participation in selected CCIEH by regions is as follows: (a) northern Europe, 35%; (b) southern Europe, 22%; (c) eastern Europe, 15%; and western Europe, 78%.

### 3.2. Conceptualization and Theoretical Models Addressed in Cultural Competence Interventions in Healthcare Delivery in Europe

After conducting a content analysis of studies addressing CCIEH, four emergent categories have been identified: (a) conceptualization of the construct; (b) theoretical framework; (c) reference authors; and (d) related constructs.

In relation to the conceptualization of the construct, studies emphasize the importance of CCIEH for patients from diverse cultural backgrounds. These publications justify CCIEH by explaining and valuing the influence of culture on the delivery of healthcare to culturally diverse patients. Although in 22% of the documents the concept of CC is not defined beyond the introduction provided to the intervention program, in the remaining 78%, a definition of the term is included, either literally or through the development of the meaning of the construct via a detailed rationale of CC and its importance in the healthcare setting.

In Appendix C, the definitions of CC reported in each publication and the theoretical models are presented. It is observed that in 21% of the records, reference is made to the Campinha-Bacote model [73,74,75], in 5% to the Betancourt model [9,76,77]), and in another 11% to the Papadopoulos team model [78,79]. Also noteworthy are the models of Leininger [80], Seelman et al. [81], and Sue and Sue [82], among others. These definitions and models are detailed in Table 1.

Another notable finding is the presence of terms used interchangeably or with similar conceptualizations as CC, which were identified in 32% of the analyzed documents. Among these, it is noteworthy to mention (a) “multi” (e.g., [85]), (b) “intercultural competence” (e.g., [86]), and (c) “cross-cultural competencies” (e.g., [87]). Furthermore, other approaches derived from CC have also been introduced, providing nuances, such as (a) “cultural safety” (e.g., [88]), reflecting the need for changes in thinking about power relations and patient rights [89], (b) “competence towards diversity” (e.g., [90]), which employs the intersectionality paradigm, and (c) “cultural health capital” (e.g., [69]), which considers the competencies, attitudes, and behaviors of patients, combined with those of healthcare professionals, to achieve optimal relationships [91]. Finally, it should be highlighted that the theoretical framework framing this terminology is “transcultural nursing” (e.g., [62,92]). Transcultural nursing focuses on the study of the influence of culture on health and illness to understand current healthcare practices and contribute to their future development [93].

### 3.3. Elements and Methodological Strategies of Interventions in Cultural Competence

This section provides a detailed overview of practical aspects related to the methodology of intervention programs on cultural competence.

#### 3.3.1. Description of the Methodology of Cultural Competence Interventions

The results of the analysis of the participants (practicing professionals and students undergoing training in the healthcare sector) and target group, research design, assessment instruments, and measured variables employed are presented.

Firstly, regarding the professionals participating in the CCIEH, 28 interventions were carried out with participants from a single discipline (see Appendix D): 19 interventions in nursing; 3 in mental health; 5 in general medicine; and 1 in dentistry services. On the other hand, 26 CCIEH were implemented with multidisciplinary teams. The nursing field stood out, being represented in 88% of multidisciplinary CCIEH. Additionally, participants came from an academic context in 31% of CCIEH: these included undergraduate and postgraduate students in nursing, dentistry, and social sciences (see Appendix C). Finally, among the selected studies, the figure of the CHW (Community Health Worker) is introduced [94], a frontline public health worker who is a trusted member of and/or has an unusually close understanding of the community served. Their role is to serve as an intermediary between health/social services and the community to facilitate access to services and improve the quality and cultural competence of service delivery.

In terms of the number of professionals attending the CCIEH, the sample was highly heterogeneous: the largest group consisted of 656 nursing psychology, nursing, and dentistry students, while the smallest sample comprised eight students. Thus, nearly half of the CCIEH included a maximum of 50 individuals and only 14% developed CCIEH with large samples of over 200 participants.

Secondly, the analyzed studies specify their target population as follows: (1) primarily individuals from distinct racial/ethnic backgrounds or patients of other nationalities (81%); (2) patients considered culturally diverse (15%); and (3) population belonging to the LGBTQ+ community (4%).

Thirdly, of the total selected publications, 32% included quantitative methodology, 20% included a qualitative methodology, and the remaining 48% included a combination of both methods. With respect to the design of quantitative studies, 24% of the publications have consisted of trials with an intervention group and a control group, four of which were randomized controlled studies.

Fourthly, self-reported assessment instruments predominated in the different CCIEH, with numerous ad hoc designed questionnaires and scales (see Appendix E). Meanwhile, an additional 12 questionnaires and scales validated were utilized. Regarding qualitative methodology, the content of participants’ reflections and behaviors was thoroughly examined using diaries, direct observation techniques, and analysis of discussion groups, meetings, and focus group interview dynamics. Alternatively, objective measures were obtained through performance tests (exercises designed with vignettes and case scenarios) and the collection of administrative data (e.g., scope, fidelity, and cost-effectiveness of the CCIEH).

Finally, Table 2 displays the variables studied in relation to CC, categorized as individual variables, intervention variables, contextual variables, or follow-up variables. These variables have been analyzed to structure the factors associated with CC and to identify those aspects that receive greater emphasis in the CCIEH. In this way, certain areas have been identified in which little or no emphasis has been placed, such as group, contextual, and organizational variables.

#### 3.3.2. Description of Cultural Competence Intervention Programs

Next, the results on the duration of the CCIEH, main components, content implementation systems, intervention locations, program development techniques, and performance tests are presented.

Firstly, concerning the duration of the CCIEH (see Appendix D), workshops ranging from one-day sessions to curriculum programs developed over 3 years have been identified, although the majority of CCIEH were delivered within a timeframe of less than one week (39%). With regard to the organization of CCIEH, two different modalities have been distinguished: (a) interventions structured over a series of hours and/or modules, delivered continuously according to a pre-established agenda; and (b) CCIEH implemented flexibly over a period of time, with intervals dedicated to individual activities and reflection, assimilation and consolidation of learning.

Secondly, the content of the CCIEH has predominantly been structured around the classic components accepted by various models of cultural competence: attitudes/consciousness, cultural knowledge, and cultural skills. At times, additional components have been added to these elements, in line with the conceptual approaches guiding the CCIEH (e.g., cultural sensitivity according to the Campinha-Bacote model [73]).

Thirdly, with respect to the implementation systems of CCIEH content, 15% have utilized online digital technologies and social networks. Besides, six of these interventions have been developed entirely through the internet. Additionally, the use of these technologies received positive feedback from participants and the training team, appreciating the advantages of distance learning and adaptability to the nature of healthcare activities and their work schedules (e.g., [95]).

Fourthly, CCIEH were conducted in various locations, including universities, healthcare centers, hospital units, mental health services, nursing homes, prenatal care centers, maternity ward, residential care units for children and youth, municipal facilities and even in a mosque. In most cases, the professionals’ regular workplace was chosen to avoid commuting and to accommodate their work schedules.

Fifthly, a wide variety of techniques have been used for the development of CCIEH programs: lectures, workshops and seminars, independent reading of material, individual and group practical exercises, reflections, debates, presentations, videos, participant observation, simulation exercises through role-playing games (including professional actors), online learning tasks, questionnaires, objective performance tests (e.g., vignettes), and clinical case studies. Several studies highlighted the effectiveness of techniques that allowed the application of learned skills in practical contexts, such as the use of role-playing with simulated patients, compared to more traditional approaches (e.g., [96]).

Lastly and sixthly, several CCIEH included data obtained from patients among the measures used to evaluate the outcomes. The results obtained from the evaluations report several benefits derived from CCIEH: (a) changes in healthcare and communicative behavior after intervening with both healthcare professionals and patients (e.g., [58,59]); (b) satisfaction interviews with recipients of healthcare services (e.g., [69,97]); (c) analysis of narratives emerging during consultations (e.g., [66,67]); (d) observation of mood, cooperation, and delays caused by the patient (e.g., [87]); (e) pregnant women’s health literacy levels (e.g., [72]); (f) reports on the level of observed cultural competence in healthcare providers (e.g., [98]); and (g) perinatal mortality and morbidity outcomes (e.g., [71]).

## 4. Discussion

The objective of this study has been to map the scientific literature focused on interventions aimed at enhancing «cultural competence» among healthcare professionals in Europe. To achieve this, a qualitative exploratory systematic review, also known as a scoping review, was conducted.

Regarding the first research objective, focused on the scientific production and geographical distribution of interventions, the data reflect a moderate interest in the topic. Although there has been a gradual increase in the number of identified publications in recent years, the volume of selected documents has remained relatively low, ranging between zero and four until 2019. It is worth noting that 36% of the publications have been produced in the last four years. On the other hand, the limited number of CCIEH in countries in eastern, central, and southern Europe has been a significant finding of our review. Despite the scarce literature in this field, our results suggest a notable lack of research in these regions compared to other parts of Europe.

These findings underscore the need for greater attention and development of CCIEH in underrepresented geographical contexts, which may be crucial for addressing the challenges of cultural diversity in healthcare education. Thus, despite the increasing migration flows in recent years and the growing cultural diversity [1], less than half of the European countries (23 out of 50) have conducted significant research on the contribution of healthcare education to cultural competence. Among these, 35% have carried out only a single intervention. These data are particularly relevant as they highlight the necessity for increased attention and effort in this field of study in Europe.

The limited presence of interventions in Mediterranean countries is notable, despite the region’s significance as a key point on the African migration route to Europe. Additionally, certain countries (e.g., France and Belgium) have historically extended their cultural influence, particularly linguistically, worldwide during the 19th and 20th centuries and have received culturally and historically related migrants in recent decades. However, their contribution to research on cultural competence represents only 5% of the publications, with participation primarily in multinational projects.

In contrast, a higher number of interventions have been recorded in the Netherlands (8 publications), Sweden (10), and the United Kingdom (21), suggesting a need for a deeper analysis to understand the underlying reasons for this greater demand. This interest in the topic may stem from numerous social factors that could be contributing to fostering research in these countries. For example, in the United Kingdom, there has been recognition of the need to address cultural issues in healthcare since the late 1990s. Consequently, efforts to promote training in cultural competence for healthcare professionals began through the implementation of healthcare policies and professional guidelines, such as those from the National Institute for Health and Care Excellence (NICE) [99,100]. On the other hand, for the assimilationist model in France, ethnic minorities were considered social identities in the process of converging with the majority culture. For years, a model of the French citizen corresponding to the republican ideal was encouraged, characterized by a single cultural identity. As a result, ideas of republicanism and uniformitarianism caused cultural identities to be suppressed and not valued [101,102,103]. This could justify the lack of legal, political, and academic interest in cultural diversity, migrant integration, and the development of intercultural competencies in the healthcare field [104,105]. Other examples of assimilative practices are the discourses developed in the dialectical process of identity construction [106].

On the other hand, in Germany, assimilationism carries more negative connotations due to its association with historical policies of forced Germanization, resulting in a rejection of any approach suggesting cultural coercion. Germany has maintained institutionalized separation through policies such as segregated education and the provision of social services through charity organizations based on religious or political affiliations. These practices perpetuate distinctions and reject approaches, suggesting cultural coercion [107]. Although Germany has begun to change its policies to promote both cultural assimilation and economic integration of immigrants [108], the legacy of assimilationist practices continues to influence the lack of development of cultural competence in the healthcare field. This could explain why cultural competence has not been a widely researched topic, unlike in other European countries where cultural diversity is addressed with more openness and pluralism.

In relation to the second objective, which concerns the conceptualization of CC, it was found that 78% of the interventions included a definition of the construct and/or related concepts. Although the term CC was used in 35% of the interventions that provided a conceptual definition, the remaining studies employed related concepts to express nuances or expand the terminological meaning. Nevertheless, some of the terms have been used synonymously, ultimately as adjectives of competence (cross-cultural, intercultural, and multicultural), without clarifying the difference provided by that conceptualization compared to the classical terminology of CC and whether they represent different conceptualizations. Although the concept of CC is complex, a construct becomes ambiguous if it can be assigned more than one meaning or if the meaning is unclear in a particular context [109].

On the other hand, various sources were used as references to describe the meaning attributed to CC in healthcare (e.g., Campinha-Bacote [73] and the British research team of Papadopoulos [78]). The most commonly used models for delivering CCIEH are based on Leininger’s Theory of Transcultural Nursing Care [80]. However, some conceptualizations of CC propose a broader approach based on the development of CC that enables healthcare professionals to interact with all patients, prioritizing individual diversity over focusing on belonging to specific ethnic and social groups (patient-centered approaches). The practice of naming specific groups implies that cultural competence varies depending on the group to which the person belongs and their ethnic or racial identity, ignoring other intersecting identities. As Ridley points out [110], individuals who identify as White also have a racial and ethnic identity and a cultural context that bears consideration.

In this direction, some CCIEH have been included under the paradigm of intersectionality (e.g., [42]). The underlying objective was to develop multicultural competence that considers the interaction processes between various dimensions of individual difference, such as sex and gender, social class, race and ethnicity, sexuality, functional ability, and age, among others. As noted by Kumagai and Lypson [111], for these conceptualizations, the construct of CC extends beyond the traditional notions of changing attitudes and increasing knowledge and professional skills of a specific population group, toward an approach that encompasses all individuals and their own diversity. This implies a “critical awareness” of both oneself and others, as well as a commitment to consider all relevant social factors in the provision of healthcare.

Based on the foregoing and presence of a vast majority of interventions aimed at migrant populations or belonging to racial or ethnic groups (81%), a bias is observed in the interpretation of the definition of CC, primarily focused on a single dimension of individual identity. This could result in a limitation of the potential benefits that CCIEH could provide to society. For instance, the exclusion of other dimensions that constitute culture could affect the content of the components selected to implement CCIEH. Therefore, it would be advisable to conduct future research to clarify the evolution and development of the CC construct, the underlying theories, and the models being implemented, to provide clarity and a uniform understanding of CC.

On the other hand, the operationalization of CC was very similar in all cases. Regardless of the definition used to introduce the concept of CC, in practice, the content of the interventions revolved around a set of components: cultural awareness and sensitivity, the desire to engage with culturally diverse individuals, cultural knowledge, and the professional skills necessary to effectively navigate cultural diversity. In some of the models studied, additional dimensions can even be found (see Papadopoulos et al.’s model of compassionate care [112]). This confirms the complexity of operationalizing CC: there are various components that are not universally accepted, and it is not precisely known how they interact with each other and to what extent some influence others in the acquisition of professional CC [113].

The third research objective, focusing on the analysis of intervention characteristics, addresses various crucial aspects related to CCIEH. Firstly, it explores the notable heterogeneity of the interventions, as well as the absence of a pedagogical approach, which complicates the understanding of underlying learning models. Secondly, it examines the composition of participant samples, highlighting the prevalence of small samples and the significant presence of the nursing discipline. Thirdly, it also analyzes the participation of nursing students in these interventions, emphasizing their role in developing cultural sensitivity and applying knowledge in clinical practice. Fourthly, another point to consider is the limited representation of healthcare service users in program design, as well as the importance of their active participation in enhancing the effectiveness of CCIEH. Fifthly, it discusses the training strategies used, emphasizing the variety of approaches and the effectiveness of certain methodologies in promoting learning. Lastly, it examines the research methodology employed, noting limitations and the need for future more rigorous research to evaluate the effectiveness of these interventions.

Regarding the first point, a marked heterogeneity is observed in all the elements that make up the intervention programs, which complicates the comparison between them and hinders the extraction of conclusions about their effectiveness. In some CCIEH, only one of the components has been addressed; in others, the dimensions of the model used as a reference have been targeted and in the remaining cases, the model used for the intervention is unknown. Therefore, the conclusions about the results achieved cannot be attributed to a specific component. It is also impossible to identify which actions yield better results or, in case the intervention objectives are not met, the possible aspects to improve. Furthermore, it is possible that success is due to an interaction of the components with each other. Specific research on the effectiveness of each component separately and their interaction through a systematic literature review in this area would be necessary. This way, relevant conclusions could be reached to guide the design of other interventions, organizational practices, and government policies.

Secondly, concerning the participants in the interventions, it is noteworthy that in 44% of cases, small samples were selected (fewer than 50). Only nine interventions were conducted with samples of over 200 participants. Additionally, it is relevant to note that the discipline of nursing had a significant presence, being present in 88% of the CCIEH, demonstrating a high level of interest and greater participation of professionals in this field. In fact, 19 of the interventions were exclusively aimed at nursing professionals, while four others focused on the field of mental health, which proved to be the second most significant. As mentioned earlier, this phenomenon reflects the influence of the tradition of studying CC by Transcultural Care Theories.

Thirdly, it is observed that 31% of interventions are targeted at university students, entailing 12 recruited nursing students, including 11 undergraduate and one postgraduate students. There is an evident interest in incorporating knowledge about CC into the healthcare field from the early stages of a nursing professional’s career, especially through exchange and immersion programs in other countries. This approach not only facilitates the comparison of knowledge and clinical practices but also promotes a critical attitude and allows for experiencing cultural shock, which can foster the early development of cultural sensitivity. It would be pertinent to analyze this aspect more deeply in future research to understand at which point in the professional’s career training in CC could be most effective. In this regard, implementing longitudinal studies on the development and various demands of the professional career could provide valuable insights into how CCIEH influences the application of learning in clinical practice. This approach could offer a more comprehensive view of the effectiveness of these interventions over time and at different stages of the professional career.

Fourthly, the findings of this review demonstrate a lack of interventions aimed at professionals responsible for training healthcare personnel. Only one intervention has been identified, oriented toward mentors of nursing professionals within the university education framework (e.g., [114]). Consequently, there is a knowledge gap regarding CC among trainers themselves and the level of knowledge they possess, including models, theoretical approaches, and therapeutic skills, among other aspects. Overall, it seems that external experts are often relied upon and there is no information available regarding the training program they have received to prepare them for this role. On the other end, healthcare service users typically do not participate in the development and implementation of intervention programs. Thus, only in two selected interventions were patients’ opinions collected to evaluate the results obtained. This aspect is an area needing attention for the development of future interventions, as the active participation of program recipients designed to improve their health outcomes could contribute to defining necessary objectives, refining intervention contents, and achieving greater efficiency in results.

Fifthly, concerning the training strategies employed, interventions have varied widely in content, implementation methodologies, and duration. The suitability of digital technology and online programs for accommodating the availability of professionals’ schedules are specifically highlighted. Additionally, good results have also been emphasized with participatory problem-solving methodologies in clinical cases, role-playing, simulations, and immersion programs [115,116]. These methodologies offer learning opportunities that facilitate reflection, the development of critical thinking, and practical skills. This aspect aligns with one of the objectives outlined in various CCIEH; thus, it would be advisable to increase the utilization of such exercises in future research and assess their relative contribution to the final outcomes.

Finally, from a methodological standpoint, most of the research has been conducted using uncontrolled designs and mixed techniques, both quantitative and qualitative. Furthermore, evaluative measures were taken both before and after the intervention in two-thirds of the CCIEH. Moreover, upon analyzing the measurement instruments used, it was revealed that a significant portion of the techniques employed were created specifically for each intervention. These factors hinder the availability of research with sufficient methodological quality to draw conclusions about the effectiveness of the interventions. In this regard, it would be advisable to conduct a systematic review analyzing the most effective practices and delve deeper into what each component contributes. The heterogeneity of techniques and technologies employed the multimodal approach of most interventions and the disparity in their evaluation systems all suggest this approach.

This scoping review has limitations inherent to its extent as its aim is to provide a comprehensive overview of the scientific literature, specifically focusing on the European context, rather than conducting a detailed analysis of each individual study. Therefore, without having evaluated the bias of the included studies, it is recommended to interpret the findings with caution, restricting them to the geographical scope studied.

Another limitation concerns the breadth of this review: it is possible that there are interventions published at a local level and others that have not been reported, making them difficult to locate. However, since the analysis was conducted using international databases and the literature search was extensive, the conclusion regarding the scarcity of CCIEH could be considered valid.

Furthermore, most of the assessment tools used in the interventions are based on self-reports. However, these instruments are not reliable for predicting changes in behavior, sensitivity, and effective clinical practice [117,118,119]. It is important to highlight this point, as Larson and Bradshaw [120] have previously noted a significant association between CC and social desirability bias [121]. That is, the relationships found in attitudes, affect, and behavior could be an expression of participants’ desire to create a favorable social impression. Therefore, interventions need to be conducted to control this bias and increase the use of objective performance assessment instruments.

## 5. Conclusions

Interventions in cultural competence for healthcare professionals represent a solution to the growing and dynamic cultural and social superdiversity. These programs promote awareness and sensitivity toward the influence of culture in healthcare, providing knowledge and tools to deliver effective healthcare for all individuals in today’s society. However, the widespread adoption of such interventions has not been achieved in Europe. Furthermore, the heterogeneity of their implementations does not allow us to know if the models used have been precisely adapted to the socio-cultural needs and characteristics of Europe. Future specific studies on cultural competence focused on the European social and political context can contribute to the development of the conceptualization and operationalization of cultural competence and thus contribute to the recognition of diversity in healthcare and to social equity.

## Figures and Tables

**Table 1:** Definitions and models of cultural competence in healthcare: key references.

Authorship	Definition	Model
Campinha-Bacote [73,74,75]	The process by which healthcare professionals continuously strive to achieve the ability to work effectively within the cultural context of individuals, families, or communities of diverse cultural/ethnic backgrounds (p. 181, [73]).	Identifies five dimensions: (a) cultural awareness, (b) cultural knowledge, (c) cultural skills, (d) cultural encounters, and (e) cultural desire.
Betancourt [9,76,77]	The professional develops intercultural competencies that allow them to communicate with all patients and to be sensitivity and empathy toward their norms, beliefs, values and thought patterns of each individual (p. 5, [77]).	Three conceptual approaches: (a) attitudes (cultural sensitivity/awareness), (b) knowledge (multicultural/categorical education), and (c) skills (intercultural training).
Papadopoulos et al. [78,79]	A continuous learning process through which a set of competencies is developed at two levels. Culturally generic competencies are acquired, leading to the development of specific cultural competencies. These, in turn, feed into the expansion of generic competencies in a spiral process, enabling the professional to work effectively within the patient’s context (p. 6, [79]).	Each level includes four interconnected elements: (a) cultural awareness, (b) knowledge, (c) sensitivity, and (d) cultural competence.
Leininger [83]	Transcultural Care Theory defines cultural care as the learned and transmitted cognitive values, beliefs, and lifestyle patterns of both professional and indigenous groups, which are used to assist, facilitate, or enable another individual or group to maintain their well-being or health or to improve a human condition or way of life. The culturally competent professional is capable of assessing and understanding culture, care, and health factors and using this knowledge creatively with people of diverse or similar lifeways (p. 117, [84]). The theory of cultural care is represented by the Sunrise Model.	It comprises six main domains: (a) worldview of cultural care, (b) dimensions of cultural and social construction, (c) diverse healthcare systems, (d) nursing care decisions and actions, (e) modalities of cultural care, and (f) congruence of cultural care.
Sue and Sue [82]	Dynamic process of becoming aware and recognizing individual and cultural differences. It builds the healthcare relationship by considering the client, the healthcare provider, and the context [82].	Composed of three necessary and interrelated dimensions: (a) awareness, (b) knowledge, and (c) skills.
Seeleman et al. [81]	A learning process that emphasizes various relevant aspects in the delivery of healthcare to diverse ethnic groups. It demonstrates that there are more dimensions than merely cultural to achieve high-quality healthcare provision. It proposes developing specific knowledge about various ethnic groups (e.g., epidemiology), becoming aware of how culture determines individual behavior and thought, and acquiring the ability to convey information, seek external assistance, and adapt to new situations [81].	It illustrates three cultural competencies: (a) knowledge, (b) awareness, and (c) ability.

**Table 2:** Variables studied related to cultural competence.

Variables	Cognitive Competence	Affective Competence	Behavioral Competence	Reflective Competence
Individual Variables	Cultural knowledge about target populations. Cultural Intelligence.	Prejudiced attitudes and stereotypes. Cultural anxiety. Desire and cultural encounters.	Self-assessment of cultural clinical skills (relationship, communication, assessment, and intervention skills). Cultural Competence. Cultural sensitivity and awareness. Perspective-taking.	
Intervention Variables	Content. Health literacy.	-	-	Reflection on the qualitative impact of training on professional practice.
Contextual Variables	-	-	Barriers, challenges, and facilitators in cultural care and treatment.	-
Follow-up Variables	-	-	-	Perceived need for further training. Previous training in CC. Work experience. Reasons for participating in the interventions.

Note: Variables such as age, gender, personality traits, gender ideology, intervention methodologies and media, organization and facilities, trainers, duration, positive aspects and areas for improvement, overall satisfaction, and perceived benefits have also been addressed.

## Data Availability

Data Availability Statements are available in “OSF Registrations” at https://archive.org/details/osf-registrations-wjh6y-v1 (accessed on 17 May 2023).

## Supplementary Materials

The following supporting information can be downloaded at: https://www.mdpi.com/article/10.3390/healthcare12101040/s1, Table S1. Search string in specific database.

## Appendix A

**Table A1:** Preferred Reporting Items for Systematic Reviews and Meta-Analyses Extensions for the Scoping Reviews (PRISMA-ScR) Checklist [47].

Section	Item	PRISMA-ScR Checklist Item	Reported on Page #
Title			
Title	1	Identify the report as a scoping review.	1
Abstract			
Structured summary	2	Provide a structured summary including, as applicable, background, objectives, eligibility criteria, sources of evidence, charting methods, results, and conclusions that relate to the review question(s) and objective(s).	1
Introduction			
Rationale	3	Describe the rationale for the review in the context of what is already known. Explain why the review question(s)/objective(s) lend themselves to a scoping review approach.	1–4
Objectives	4	Provide an explicit statement of the question(s) and objective(s) being addressed with reference to their key elements (e.g., population or participants, concepts, and context) or other relevant key elements used to conceptualize the review question(s) and/or objective(s).	4
Methods			
Protocol, and registration	5	Indicate if a review protocol exists, if and where it can be accessed (e.g., web address), and, if available, provide registration information including registration number.	5
Eligibility criteria	6	Specify the characteristics of the sources of evidence (e.g., years considered, Language and publication status) used as criteria for eligibility and provide a rationale.	5–6
Information sources	7	Describe all information sources (e.g., databases with dates of coverage, contact with authors to identify additional sources) in the search, as well as the date the most recent search was executed.	6
Search	8	Present the full electronic search strategy for at least one database, including any limits used, in order that it could be repeated.	6
Selection of sources of evidence	9	State the process for selecting sources of evidence (i.e., screening and eligibility) included.	6
Data charting process	10	Describe the methods of charting data from the included sources of evidence (e.g., piloted forms, forms that have been tested by the team before their use, whether data charting was carried out independently, in duplicate) and any processes for obtaining and confirming data from investigators.	6, 7
Data items	11	List and define all variables for which data were sought and any assumptions and simplifications made.	6, 7
Critical appraisal of individual sources of evidence	12	If performed, provide a rationale for conducting a critical appraisal of included sources of evidence, describe the methods used, and how this information was used in any data synthesis (if appropriate).	N/A
Summary measures	13	Not applicable for scoping reviews.	
Synthesis of results	14	Describe the methods of handling and summarizing the data that were charted.	7
Risk of bias across studies	15	Not applicable for scoping reviews.	
Additional analyses	16	Not applicable for scoping reviews.	
Results			
Selection of sources of evidence	17	Give numbers of sources of evidence screened, assessed for eligibility, and included in the review, with reasons for exclusions at each stage, ideally using a flow diagram.	7, 8
Characteristics of sources of evidence	18	For each source of evidence, present characteristics for which data were charted and provide the citations.	9–14
Critical appraisal within sources of evidence	19	If performed, present data on critical appraisal of included sources of evidence (see item 12).	
Results of individual sources of evidence	20	For each included source of evidence, present the relevant data that were charted that relate to the review question(s) and objective(s).	Appendix B, Appendix C, Appendix D and Appendix E
Synthesis of results	21	Summarize and/or present the charting results as they relate to the review question(s) and objective(s).	7–14
Risk of bias across studies	22	Not applicable for scoping reviews.	
Additional analyses	23	Not applicable for scoping reviews.	
Discussion			
Summary of evidence	24	Summarize the main results (including an overview of concepts, themes, and types of evidence available), explain how they relate to the review question(s) and objectives, and consider the relevance to key groups.	14–17
Limitations	25	Discuss the limitations of the scoping review process.	17–18
Conclusions	26	Provide a general interpretation of the results with respect to the review question(s) and objective(s), as well as potential implications and/or next steps.	18
Funding			
Funding	27	Describe sources of funding for the included sources of evidence, as well as sources of funding for the scoping review. Describe the role of the funders of the scoping review.	18

## Appendix B

**Table A2:** Geographical Distribution of Cultural Competence Interventions Targeting European Healthcare Professionals.

Authorship	Country
Ekblad et al., 2000 [122]	SE
Scholes and Moore, 2000 [92]	BG, NL, ES
Chevannes et al., 2002 [123]	UK
Webb and Sergison, 2003 [124]	UK
Papadopoulos et al., 2004 [125]	UK
Salas et al., 2004 [97]	UK
Sandin et al., 2004 [126]	SE
Harmsen et al., 2005 [59]	NL
Krajic et al., 2005 [127]	AT, FR, DE, IE, IT, ES, SE
Schouten et al., 2005 [58]	NL
Thomas and Cohn, 2006 [128]	UK
Gebru et al., 2008 [62]	SE
Hutnik and Gregory, 2008 [129]	UK
Papadopoulos et al., 2008 [130]	UK
Kelly and Papadopoulos, 2009 [65]	UK
Berlin et al., 2010 [131]	SE
Beune et al., 2010 [60]	NL
Gebru and Willman, 2010 [63]	SE
Beune et al., 2011 [61]	NL
Elsegood and Papadopoulos, 2011 [64]	UK
Luger [132]	UK
Moleiro et al., 2011 [133]	PT
Ascoli et al., 2012 [66]	UK
Celik et al., 2012 [134]	NL
Prescott-Clements et al., 2013 [135]	UK
Stone et al., 2013 [136]	UK
Bäärnhielm et al., 2014 [137]	SE
Moleiro et al., 2014 [85]	PT
Owiti et al., 2014 [68]	UK
Bhui et al., 2015 [67]	UK
Dowling et al., 2015 [138]	IE
Hovland and Johannessen, 2015 [139]	NO
Paroz et al., 2016 [96]	CH
Shea et al., 2016 [140]	CY
Pereira et al., 2017 [141]	PT
Ulvund and Mordal, 2017 [142]	NO
Sempértegui et al., 2018 [90]	NL
Kaihlanen et al., 2019 [143]	FI
Nielsen et al., 2019 [144]	DK
von Lersner et al., 2019 [86]	DE
Donisi et al., 2020 [145]	LT, UK, BE, IT, PL, BG
Filmer and Herbig, 2020 [87]	DE
Johnsen et al., 2020 [146]	DK
Miah et al., 2020 [147]	UK
van der Giessen et al., 2020 [148]	NL
Damsted Rasmussen et al., 2021 [70]	DK
Fair et al., 2021 [149]	GR, NL, UK
Granel et al., 2021 [150]	AT, HR, UK, FI, HU, ES, CH, BE, CZ, EE, DE, SE, NL
Johnsen et al., 2021 [69]	DK
Leung et al., 2021 [151]	SE, AU, HK
Majda et al., 2021 [152]	PL
McDonald et al., 2021 [153]	SE
Prosser et al., 2021 [154]	UK
Alarcao et al., 2022 [95]	PT
De Diego-Cordero et al. 2022 [88]	ES
Hoens et al., 2022 [94]	BE
Oikarainen et al., 2022 [114]	FI
Sánchez De Miguel et al., 2022 [155]	ES
Skoog et al., 2022 [156]	SE
Damsted Rasmussen, Fredsted Villadsen et al., 2023 [71]	DK
Damsted Rasmussen, Nybo Andersen et al., 2023 [72]	DK
Deshmukh et al., 2023 [98]	UK
Skjerve et al., 2023 [157]	NO

Note: Countries (ISO-3166-1 ALPHA-2) [158]: AT (Austria), AU (Australia), BE (Belgium), BG (Bulgaria), CH (Switzerland), CZ (Czech Republic), CY (Chipre), DE (Germany), DK (Denmark), EE (Estonia), ES (Spain), FI (Finland), FR (France), GR (Greece), HK (Hong Kong), HR (Croatia), HU (Hungary), IE (Ireland), IT (Italy), LT (Lithuania), NL (Netherlands), NO (Norway), PL (Poland), PT (Portugal), SE (Sweden), and UK (United Kingdom).

## Appendix C

**Table A3:** Definition and Models of the Retrieved Records.

d	Definition	Model	Based on
Ekblad et al., 2000 [122].	Cultural Competence Campinha-Bacote [74].	Multicultural end-of-Life Care Model.	Campinha-Bacote’s Model [74].
Scholes and Moore, 2000 [92].	Not included. Culturally Sensitive Care Campinha-Bacote [74]. Transcultural Nursing Theory Leininger, 1995 [93].	Exchange Program Model.	Leininger Transcultural Theory and Sunrise Model [93].
Chevannes et al., 2002 [123].	Not included. Transcultural Nursing Theory Leininger [159].	Transcultural Nursing Theory.	Leininger’s Transcultural Theory and Sunrise Model [159].
Webb and Sergison, 2003 [124].	Cultural Competence Dillard et al. [160]; Zayas et al. [161].	Cultural Competence Antiracism Training Model.	No information.
Papadopoulos et al., 2004 [125].	Cultural Competence Papadopoulos [112].	Culturally Compassionate Health Care Model.	Papadopoulus et al. Model (1998) [78].
Salas et al., 2004 [97].	No information.	Culturally Sensitive Clinical Service.	No information.
Sandin et al., 2004 [126].	Cultural Competence Campinha-Bacote [73].	Exchange Program Model.	Campinha-Bacote’s model [73]; Transcultural Nursing Theories Andrews and Boyle [162]; Leininger and McFarland [84]
Harmsen et al., 2005 [59].	Not included. Intercultural Communication Pinto [163]. Explanatory Models Kleinman [164].	Intercultural Communication.	Pinto’s Theory [163].
Krajic et al., 2005 [127].	Cultural Competence Cross et al. [21].	Cultural Competence Model.	Campinha-Bacote’s Model [73].
Schouten et al., 2005 [58].	Cultural Competence Brach and Fraser [33]; Carrillo et al. [165]; and Kagawa-Singer and Kassim-Lakha [166] Explanatory models Kleinman [164].	Intercultural Communication Theory.	Pinto’s Theory [163].
Thomas and Cohn, 2006 [128].	No information.	Communication Skills and Cultural Awareness Training Model.	Modification of the Cancer Research UK advanced Communication Skills Training Model. Fallowfield [167].
Gebru et al., 2008 [62].	Not included. Transcultural Nursing Theory Leininger [93].	Research-Based Didactic Model for Transcultural Nursing.	Leininger Transcultural Theory and Sunrise Model [93].
Hutnik and Gregory, 2008 [129].	Cultural Competence Campinha-Bacote [74].	Cultural Competence Model.	Camphina-Bacote’s Model [74].
Papadopoulos et al., 2008 [130].	Cultural Competence Papadopoulos [79].	Culturally Compassionate Health Care Model.	Papadopoulus et al. Model [79].
Kelly and Papadopoulos, 2009 [65].	Cultural Competence Cross et al. [21]. Betancourt et al. [76].	Culturally Compassionate Health Care Model.	Papadopoulus et al. Model [78].
Berlin et al., 2010 [131].	Cultural Competence Campinha-Bacote [73].	Cultural Competence Model.	Campinha-Bacote’s Model [73].
Beune et al., 2010 [60].	No information.	Culturally Sensitive Counselling and Culturally Appropriate Care.	Patient-centered Educational Approaches (no author information).
Gebru and Willman, 2010 [63].	Cultural Competence Leininger [84].	Research-based Didactic Model For Transcultural Nursing.	Leininger Transcultural Theory and sunrise Model [93].
Beune et al., 2011 [61].	No information.	Culturally Appropriate Patient-Centered Educational Approaches.	Patient-Centered Approach Kleinman et al. [168].
Elsegood and Papadopoulos, 2011 [64].	Cultural Competence Cross et al. [21]; Papadopoulos et al. [78].	Culturally Compassionate Health Care Model.	Papadopoulus et al. Model [78].
Luger [132].	Cultural Competence O’Hagan, 2001 [169].	Culturally Sensitive Model.	Campinha-Bacote’s Model 2003.
Moleiro et al., 2011 [133].	Cultural Competence Sue [170]. Multicultural counseling competence. Sue et al. [171].	Cultural Diversity Competence Program.	Cultural Competence Model Sue [170]. Multicultural Counseling Competence. Sue et al. [171].
Ascoli et al., 2012 [66].	Cultural Competence Cross et al. [21]; Mareasa and Marva [172]; Sue et al. [171]; Davis [173].	Cultural Consultation Service (CCS) Model.	McGill model. Kirmayer et al. [174].
Celik et al., 2012 [134].	Not included. Cultural Diversity Appropiate Care Leininger [175].	Awareness training to promote diversity sensitivity model.	Diversity Sensitivity Models Bekker [176]; Moser [177]; Pinn [178]; Van Mens-Verhulst [179]. Nursing theories Leininger [93]; Mahoney and Engebretson [180].
Prescott-Clements et al., 2013 [135].	Cultural Competence Betancourt [9].	Patient-Centered Cultural Approach.	Betancourt’s Model [9].
Stone et al., 2013 [136].	No information.	Culturally Compassionate Health Care Model.	Papadopoulus et al. Model [78].
Bäärnhielm et al., 2014 [137].	No information.	Cross-Cultural Mental Health.	No information.
Moleiro et al., 2014 [85].	Not included. Multicultural Competence Sue et al. [171]	Multicultural Counseling LGB(T) Training Model.	Sue et al. Model [171]. Israel and Selvidge Model [181].
Owiti et al., 2014 [68].	Cultural Competence Cross et al. [21]; Mareasa and Marva [172]; Sue et al. [171]; Davis [173].	Cultural Consultation Service (CCS) Model.	McGill model. Kirmayer et al. [174].
Bhui et al., 2015 [67].	Not included. Cultural Consultation Kirmayer et al. [174].	Cultural Consultation Service (CCS) model.	McGill Model. Kirmayer et al. [174].
Dowling et al., 2015 [138].	No information.	Elective program.	No information.
Hovland and Johannessen, 2015 [139].	Cultural Competence Campinha-Bacote [182]. Cultural Sensitivity Hughes and Hood [183].	Exchange Program, focused on culture and global/international health.	Campinha-Bacote’s Model [73].
Paroz et al., 2016 [96].	Cultural Competence Betancourt [184].	Simulated Clinical Encounter in a Cultural Competence Education Module.	Betancourt’s Model [184].
Shea et al., 2016 [140].	No information.	Compassionate care.	Shea et al. Model 2014 [185].
Pereira et al., 2017 [141].	No information.	Exchange Program on Culturally Sensitive Care and Community Health.	No information.
Ulvund and Mordal, 2017 [142].	Cultural Competence Papadopoulos [79].	Exchange Program.	Papadopoulus et al. Model [78].
Sempértegui et al., 2018 [90].	Cultural Competence Kirmayer [186]. Diversity Competence Bechtel and Ness [187]. Intersectionality McCall [42]; Van Mens-Verhulst and Radtke [188].	Diversity-Oriented Approach.	Intersectionality Model Collins [189]; Crenshaw [190]; McCall [42]; Van Mens-Verhulst and Radtke [188].
Kaihlanen et al., 2019 [143].	Cultural Competence Campinha-Bacote [73].	Cultural Competence Model.	Campinha-Bacote’s Model [73].
Nielsen et al., 2019 [144].	Cultural Competence Campinha-Bacote [75]; Papadopoulos et al. [125].	Culturally Compassionate Health Care Model.	Papadopoulos et al. Model [78].
von Lersner et al., 2019 [86].	Not included. Intercultural Competence Sue and Sue [82].	Intercultural competence Diversity approach.	Sue and Sue Model [82]. Van Keuk et al. Model [191].
Donisi et al., 2020 [145].	Cultural Competence Boroughs et al. [192].	Health4LGBTI Cultural Competence Training Model.	Boroughs et al. Model [192].
Filmer and Herbig, 2020 [87].	Cultural Competence. Campinha-Bacote [73]; Betancourt [193]. Cross-Cultural Competencies Betancourt [193]; Kleinman and Benson [36].	Cross-Cultural Communication Model.	Campinha-Bacote’s Model [73]; Taylor-Ritzler et al. [194].
Johnsen et al., 2020 [146].	Cultural Competence Seeleman et al., 2009 [81] Cultural Health Capital Shim, 2010 [195].	Intercultural Communication and Cultural Competence Model.	Seeleman et al. Model [81].
Miah et al., 2020 [147].	No information.	Diversity training.	No information.
van der Giessen et al., 2020 [148].	Cultural Competence Sorensen et al. [196].	Culturally Sensitive Communication Model.	Limited Health Literacy Care. Sudore and Schillinger [197].
Damsted Rasmussen et al., 2021 [70].	Not included. Cultural Health Capital Dubbin et al. [91].	Intercultural Communication and Cultural Competence Model.	Seeleman et al. Model [81] Cultural Health Capital. Dubbin et al. [91] Health literacy. Osborne et al. [198].
Fair et al., 2021 [149].	Cultural Competence Seeleman et al. [81].	Culturally Sensitive Maternity Care Model.	Seeleman et al. Model [81].
Granel et al., 2021 [150].	Cultural Competence Campinha-Bacote [73]; Harkess and Kaddoura [199]. Intercultural Competence Savicki [200].	Exchange Programs: European Network of Nursing in Higher Education (ENNE).	Campinha-Bacote’s Model [73]; Intercultural Competencies Model Savicki [200]. Patient Centered Care Darnell and Hickson [201].
Johnsen et al., 2021 [69].	Not included. Cultural Health Capital Dubbin et al. [91].	Intercultural Communication and Cultural Competence Model.	Seeleman et al. Model [81].
Leung et al., 2021 [151].	Cultural Competence Betancourt et al. [9,76].	Cultural Awareness Model.	Campinha-Bacote’s Model [73]; Rew et al. Model [202].
Majda et al., 2021 [152].	Cultural Competence Camphina-Bacote 2002 [73]; Purnell 2005 [203].	Transcultural Nursing.	Giger and Davidhizar model [204].
McDonald et al., 2021 [153].	No information.	Comprehensive Cross-Cultural Training. Intercultural Communication Model.	No information.
Prosser et al., 2021 [154].	Not included. Intercultural Competence Deardorff. 2006 [205].	Transcultural psychiatry peer e-learning.	No information.
Alarcao et al., 2022 [95].	Cultural Competence Sue [206]. Multicultural Competence Chao et al. [207]. Intersectionality. McCall [42].	Multicultural Counseling Competence Model. Intersectionality Framework.	Sue and Sue’s Model [206].
De Diego-Cordero et al. 2022 [88].	Cultural Competence Kumagai and Lypson, 2009 [111]. Cultural Safety. Papps and Ramsden [89]; Curtis et al. [208].	Cultural Safety Care Model Intersectionality Model.	Papps and Ramsden [89].
Hoens et al., 2022 [94].	Cultural Competence Sharifi et al., 2019 [209].	Innovative neighbourhood care model.	Sharifi et al. (2019) model of cultural competence [209].
Oikarainen et al., 2022 [114].	Cultural Competence Garneau and Pepin [210] Campinha-Bacote [211].	Culturally Conscious Model of Mentoring.	Campinha-Bacote’s model [211].
Sánchez De Miguel et al., 2022 [155].	Not included. Invisible care. Feo and Kitson, 2016 [212]; Huercanos-Esparza, 2010 [213].	Equity and Cultural Diversity care.	Invisible care approach Feo and Kitson, 2016 [212]; Huercanos-Esparza, 2010 [213].
Skoog et al., 2022 [156].	No information.	Cultural Competence Model. Client-centered Therapy Approach.	Campinha-Bacote’s Model [73]. Rogers´Model [214].
Damsted Rasmussen, Fredsted Villadsen et al., 2023 [71].	No information.	Intercultural Communication and Cultural Competence Model.	Seeleman et al., 2009 model [81].
Damsted Rasmussen, Nybo Andersen et al., 2023 [72].	Cultural Competence Seeleman et al. 2009 [81].	Intercultural Communication and Cultural Competence Model.	Seeleman et al., 2009 model [81]. Health literacy (Trezona et al., 2017) [215].
Deshmukh et al., 2023 [98].	Cultural Competence Beach et al., 2005 [32].	Customized culturally sensitive intervention rheumatology program.	No information.
Skjerve et al., 2023 [157].	No information.	Skill training through simulations.	No information.

## Appendix D

**Table A4:** Methodological Characteristics (Disciplinary Backgrounds of Participants, Sample Size, and Approach) and Characteristics of the Interventions (Duration).

Authorship	Healthcare Discipline	Sample	Methodology	Duration
Ekblad et al., 2000 [122]	NU, SW, PH, GM, AP	76	Qualitative	3 D
Scholes and Moore, 2000 [92]	NUS	79	Mix methods	3 Mon
Chevannes et al., 2002 [123]	OT, PH, MS, NU, PR, AUX	22	Mix methods	10 Wk
Webb and Sergison, 2003 [124]	GM, NU, PSY, AUX, AP	134	Quantitative	1 D
Papadopoulos et al., 2004 [125]	SW, PS, PSY, NU, AP, OT	35	Quantitative	4 Mon
Salas et al., 2004 [97]	PS, PSY, AP, GM, NU, OT, PR	139 ^a^	Mix methods	1 D
Sandin et al., 2004 [126]	NUS	8	Qualitative	3 Wk
Harmsen et al., 2005 [59]	GM	38 ^a^	Mix methods	2 Wk
Krajic et al., 2005 [127]	GM, MS, NU, AUX	143	Mix methods	10 Wk
Schouten et al., 2005 [58]	GM	38 ^a^	Mix methods	2 Wk
Thomas and Cohn, 2006 [128]	NU, GM, PSY, PH, PR	47	Quantitative	3 D
Gebru et al., 2008 [62]	NUS	157	Quantitative	3 Yr
Hutnik and Gregory, 2008 [129]	NUS	350	Mix methods	1 D
Papadopoulos et al., 2008 [130]	SW, PS, PSY, NU, AP, OT	47	Quantitative	2 D
Kelly and Papadopoulos, 2009 [65]	PNS, EPS	5	Mix methods	8 Wk
Berlin et al., 2010 [131]	NU	51	Quantitative	4 Wk
Beune et al., 2010 [60]	NU, GM	82	Quantitative	2 D
Gebru and Willman, 2010 [63]	NUS	157	Mix methods	3 Yr
Beune et al., 2011 [61]	NU, GM	16	Qualitative	2 D
Elsegood and Papadopoulos, 2011 [64]	GM, PSY. PS, NU, SW	22	Qualitative	8 Wk
Luger [132]	NU, SW	73	Mix methods	3 D
Moleiro et al., 2011 [133]	PSY, SW, AUX	30	Quantitative	3 D
Ascoli et al., 2012 [66]	NU, PS, PSY, SW, OT	100	Qualitative	18 Mon
Celik et al., 2012 [134]	NU, PS, PSY, SW, GM, AP, AUX	31	Mix methods	4 D
Prescott-Clements et al., 2013 [135]	PDS	91	Quantitative	1 Wk
Stone et al., 2013 [136]	GM, NU, MS, AP, AUX	44	Mix methods	2 D
Bäärnhielm et al., 2014 [137]	SW, NU, PSY, GM	278	Mix methods	3 Wk
Moleiro et al., 2014 [85]	PSY	20	Mix methods	2 Wk
Owiti et al., 2014 [68]	NU, PS, PSY, SW, OT	94	Mix methods	18 Mon
Bhui et al., 2015 [67]	NU, PS, PSY, SW, OT	94 ^a^	Mix methods	18 Mon
Dowling et al., 2015 [138]	GMS	32	Qualitative	4 Mon
Hovland and Johannessen, 2015 [139]	NUS	197	Qualitative	12 Wk
Paroz et al., 2016 [96]	GM	100	Mix methods	1 D
Shea et al., 2016 [140]	NU. GM, AP	65	Mix methods	6 Mon
Pereira et al., 2017 [141]	NUS	16	Mix Methods	9 Wk
Ulvund and Mordal, 2017 [142]	NUS	18	Qualitative	4 Wk
Sempértegui et al., 2018 [90]	PS, PSY, SW	40	Quantitative	2 D
Kaihlanen et al., 2019 [143]	NU	20	Qualitative	4 Wk
Nielsen et al., 2019 [144]	NU, MS, SW, GM	30	Qualitative	6 Mon
von Lersner et al., 2019 [86]	PSY	77	Quantitative	3 Wk
Donisi et al., 2020 [145]	NU, GM, PSY, SW, PR, PH, AUX	110	Quantitative	2 D
Filmer and Herbig, 2020 [87]	NU	214	Mix methods	6 Mon
Johnsen et al., 2020 [146]	NU	18	Mix methods	1 D
Miah et al., 2020 [147]	GMS	223	Mix methods	1 D
van der Giessen et al., 2020 [148]	NU, SM	65	Quantitative	2 Wk
Damsted Rasmussen et al., 2021 [70]	NU	346	Mix methods	3 D
Fair et al., 2021 [149]	NU	57	Mix methods	2 D
Granel et al., 2021 [150]	NUS	150	Quantitative	1 Wk
Johnsen et al., 2021 [69]	NU	40 ^a^	Mix methods	3 D
Leung et al., 2021 [151]	PNS, PPS	18	Mix methods	3 Mon
Majda et al., 2021 [152]	NUS	130	Quantitative	2 Wk
McDonald et al., 2021 [153]	PS	248	Mix methods	1 D
Prosser et al., 2021 [154]	NUS	33	Qualitative	7 Wk
Alarcao et al., 2022 [95]	NU, GM, PD, PS, PSY, SW	100	Mix methods	4 Wk
De Diego-Cordero et al. 2022 [88]	NUS	165	Quantitative	1 Mon
Hoens et al., 2022 [94]	CHW, GM, AP	25	Qualitative	9 Mon
Oikarainen et al., 2022 [114]	NU	162	Quantitative	12 Wk
Sánchez De Miguel et al., 2022 [155]	NUS, PSS, DSS	656	Quantitative	7 Wk
Skoog et al., 2022 [156]	NU	34	Mix methods	3 Mon
Damsted Rasmussen, Fredsted Villadsen et al., 2023 [71]	NU	346 ^a^	Mix methods	3 D
Damsted Rasmussen, Nybo Andersen et al., 2023 [72]	NU	346 ^a^	Quantitative	3 D
Deshmukh et al., 2023 [98]	MS, GM, NU, PH	15 ^a^	Quantitative	90 Min
Skjerve et al., 2023 [157]	NUS	18	Qualitative	45 Min

Note: Duration: D (Day), Min (Minutes), Mon (Month), Yr (Year), Wk (Week); Discipline: AP (Administrative Personnel), AUX (Auxiliary Personnel), CHW (Community Health Workers), DSS (Undergraduate Dentistry Students), GM (General Medicine), GMS (General Medicine Students), MS (Medicine Specialists), NU (Nursing), NUS (Nursing Undergraduate Students), OT (Occupational Therapy), PD (Pediatrics), PDS (Postgraduate Dentistry Students), PH (Physiotherapy), PNS (Postgraduate Nursing Students), PPS (Postgraduate Psychology Students), PR (Pharmacy), PS (Psychiatry), PSY (Psychology), PSS (Undergraduate Psychology Students), and SW (Social Work); Sample: ^a^ Indicates the existence of patient data in the evaluation of the cultural competence intervention aimed at health professionals.

## Appendix E

**Table A5:** Assessment Instruments Employed to Evaluate Cultural Competence.

Authorship	Interviews	Psychometric Measures	Behavioral Observation
Ekblad et al., 2000 [122].	Focus group interviews.		
Scholes and Moore, 2000 [92].	Interviews and focus group interviews.	Ad hoc questionnaire.	
Chevannes et al., 2002 [123].	Semi-structured Interviews and focus group interviews Walklin’s model [216].	Ad hoc questionnaire.	
Webb and Sergison, 2003 [124].		Ad hoc questionnaire.	
Papadopoulos et al., 2004 [125].		Ad hoc questionnaire for assessing cultural competence (CCATool) based on the model of Papadopoulos et al. [78].	
Salas et al., 2004 [97].		Ad hoc questionnaire.	
Sandin et al., 2004 [126].	Interviews.		
Harmsen et al., 2005 [59].	Home Interview with patients.	Mutual Understanding Scale–MUS [217]. Quality of care through patient’s eyes–Quote-M [218].	
Krajic et al., 2005 [127].	Telephone interviews and group discussions.	Ad hoc questionnaire based on the Clinical Cultural Competency Questionnaire (CCCQ) by Like [219].	
Schouten et al., 2005 [58].	Home interview with patients.		Videotapes of doctor–patient consultations.
Thomas and Cohn, 2006 [128].		Ad hoc Questionnaire.	
Gebru et al., 2008 [62].		Ad hoc Questionnaire.	
Hutnik and Gregory, 2008 [129].	Semi-structured interviews.	Ad hoc Questionnaire.	
Papadopoulos et al., 2008 [130].		CAMHS Cultural Competence in Action Tool–CAMHS ‘CCATool’ [220].	
Kelly and Papadopoulos, 2009 [65].	Online reflective journals.	Ad hoc Questionnaire.	
Berlin et al., 2010 [131].		Clinical Cultural Competence Training Questionnaires: CCCTQ-PRE and CCCTQ-POST adapted [127].	
Beune et al., 2010 [60].		‘Resident Physicians’ Preparedness to Provide Cross-Cultural Care’ survey adapted [221].	
Gebru and Willman, 2010 [63].		Ad hoc questionnaire.	
Beune et al., 2011 [61].	Focus group interviews.		
Elsegood and Papadopoulos, 2011 [64].	Reflective journals.		
Luger [132].	Focus group interviews.	Ad hoc questionnaire; Cultural Competence Assessment Tool (CCATool) (Papadopoulos et al., 2004) [125].	
Moleiro et al., 2011 [133].		Ad hoc questionnaire developed from Holcomb-McCoy and Myers [222] with an objective measure (case vignette) adapted from Amato [223] Ad hoc grid.	
Ascoli et al., 2012 [66].	Team meetings and referrals from stakeholders.		Observations of routines and rituals.
Celik et al., 2012 [134].	Oral evaluations and interviews.	Ad hoc survey.	Observation.
Prescott-Clements et al., 2013 [135].		Ad hoc questionnaire (using actors as standardized patients across four cases).	
Stone et al., 2013 [136].	Telephone interviews.	Ad hoc questionnaires.	
Bäärnhielm et al., 2014 [137].	Focus group interviews.	Ad hoc questionnaires.	
Moleiro et al., 2014 [85].	Focus group interview.	Case vignette (as in Neufeldt et al. [224]).	
Owiti et al., 2014 [68].	Interview.	Adapted Tool S-GSE Scale [225]; Assessing Cultural Competence Training-TACCT [226].	
Bhui et al., 2015 [67].	Meetings and interviews (unstructured and semi-structured).	Global Assessment of Functioning (GAF) [227]; Scale to Assess Therapeutic Relationship (STAR) [228]; Health of the Nation Scale-HoNOS [229]; General health-EuroQol [230]; care needs (CANSAS); relationship with their clinician (STAR); Tool for Assessing Cultural Competence in Training (TACCT) [231].	Observations.
Dowling et al., 2015 [138].		Ad hoc questionnaire (open questions).	
Hovland and Johannessen, 2015 [139].	Reflective Journals.		
Paroz et al., 2016 [96].	Focus group interview.	Ad hoc questionnaire (including case scenarios).	
Shea et al., 2016 [140].		Ad hoc questionnaire.	
Pereira et al., 2017 [141].	Focus group interviews.	A revised version of Shinnamon et al. [232] Health Professions Schools in Service to the Nation (HPSISN) survey.	
Ulvund and Mordal, 2017 [142].	Semi-structured interview.		
Sempértegui et al., 2018 [90].		Ad hoc questionnaire and development of Attitude-Awareness, Skills and Knowledge Scale-AaSK [233].	
Kaihlanen et al., 2019 [143].	Semi-structured interviews.		
Nielsen et al., 2019 [144].	Semi-structured interviews.		Participant observation and field observations [234].
von Lersner et al., 2019 [86].		Multicultural Counseling Inventory Test-MCI [235]; Scale to Assess the Therapeutic Relationship in Community Mental Health Care, Clinician Version-STAR-C [228].	
Donisi et al., 2020 [145].		Ad hoc questionnaire.	
Filmer and Herbig, 2020 [87].	Interviews.	Adapted questionnaire on Cultural Competency [236]; adapted Questionnaire on Cultural Anxiety [237]; Empathy Scale [238]; Interpersonal Reactivity Index [239]; and ad hoc knowledge test (case vignette).	Development of an observational rating sheet, according to previous schemes [240]: communication behavior with patients and shift observations (nursing services).
Johnsen et al., 2020 [146].	Semi-structured interviews and dialogue meetings.		
Miah et al., 2020 [147].	Focus group interviews.	Ad hoc questionnaire.	
van der Giessen et al., 2020 [148].		Ad hoc questionnaire.	
Damsted Rasmussen et al., 2021 [70].	Dialogue meetings; focus group interviews; and in-depth individual interviews with patients; Cross-sectional survey.		Participant observations and field notes (service visits).
Fair et al., 2021 [149].	Semi-structured interviews.	Ad hoc questionnaire developed from the Cultural Competence Questionnaire [241] and the validated Groningen Reflection Ability Scale-GRAS [242].	
Granel et al., 2021 [150].		Ad hoc questionnaire.	
Johnsen et al., 2021 [69].	Informal conversations with midwives; focus group semi-structured interviews according to Kvale and Brinkmann’ s guide [243]; patient interviews.		Observations of midwifery visits and their interactions, according to Krogstrup and Kristiansen’s guide [244].
Leung et al., 2021 [151].	Online discussion forums and focus group interviews.	Culture Awareness Scale (mCAS) modified version, based on the Culture Awareness Scale-CAS [202].	
Majda et al., 2021 [152].		Cross-Cultural Competence Inventory-CCCI [245]; Cultural Intelligence Scale-CQS [246].	
McDonald et al., 2021 [153].	Focus group interviews.	Ad hoc questionnaire.	
Prosser et al., 2021 [154].	Focus group interviews: reflective writing.	Ad hoc questionnaire.	
Alarcao et al., 2022 [95].		Ad hoc Questionnaire.	
De Diego-Cordero et al. 2022 [88].		Social Class Questionnaire–CCS [247]; Prejudicial Attitude Test-TAP [248]; Stereotype Content Model–SCM [249]; Reduced Gender Ideology Scale-GIS [250].	
Hoens et al., 2022 [94].	Focus group and email interviews.		
Oikarainen et al., 2022 [114].		Mentors’ Competence Instrument-MCI [251,252]; and Mentors’ Cultural Competence Instrument (MCCI).	
Sánchez De Miguel et al., 2022 [155].		Ad hoc questionnaires.	
Skoog et al., 2022 [156].		Clinical Cultural Competence Training Questionnaire (CCCTQ-PRE) adapted from Lefevre et al. [253]; Clinical Cultural Competency Training. Questionnaire Post (CCCTQ POST) from Berlin et al. [131]; Swedish version of the General Self-Efficacy Scale-S-GSE [225].	
Damsted Rasmussen, Fredsted Villadsen et al., 2023 [71].	Nationwide register data: A composite perinatal mortality and morbidity outcome.		
Damsted Rasmussen, Nybo Andersen et al., 2023 [72].		Health Literacy Questionnaire [198].	
Deshmukh et al., 2023 [98].		Patient Reported Physician Cultural Competency-PRPCC [254]; Patient Enablement Instrument-PEI [255]; COM-B questionnaire [256].	
Skjerve et al., 2023 [157].	Reflective logbooks; evaluation forms.	Ad hoc questionnaires.	
