# Supplementary material for: Cultural Competence Interventions in European Healthcare: A Scoping Review
Source: Healthcare (Basel). 2024 May 17;12(10):1040. doi: 10.3390/healthcare12101040 (PMC11120989; doi:10.3390/healthcare12101040)
Supplement: Supplementary file 1 [file healthcare-12-01040-s001.zip › healthcare-2950493-supplementary.pdf]

Supplementary table

Search string in specific database.

Database	Search equation
Scopus	TITLE-ABS-KEY [("intercultural" OR "cultural competenc*" OR "cultural awareness" OR "cultural sensitivity" OR "cultural safety" OR "cultural diversity" OR "cultural humility" OR "cultural literacy") AND ("health care" OR "healthcare" OR "primary care" OR "hospital*" OR "health servic*") AND ("health* professional*" OR "health* provider*" OR "health* work*" OR "health* practitioner*" OR doctor* OR nurs*) AND (program* OR training* OR intervention*) AND (measur* OR evaluation* OR assess* OR outcome*) and NOT ("african american*" OR "black american*" OR indian* OR asia* OR aboriginal OR "torres strait islander*" OR "indigenous australian*" OR "first nation") and NOT ("systematic review*" OR "meta-analysis" OR "literature review*")].
Web of Science, Core Collection, Current Contents Connect, Derwent Innovations Index, Grants Index, KCI-Korean Journal Database, SciELO Citation Index	TOPIC: TITLE-ABS-KEY [("intercultural" OR "cultural competenc*" OR "cultural awareness" OR "cultural sensitivity" OR "cultural safety" OR "cultural diversity" OR "cultural humility" OR "cultural literacy") AND ("health care" OR "healthcare" OR "primary care" OR "hospital*" OR "health servic*") AND ("health* professional*" OR "health* provider*" OR "health* work*" OR "health* practitioner*" OR doctor* OR nurs*) AND (program* OR training* OR intervention*) AND (measur* OR evaluation* OR assess* OR outcome*) and NOT ("african american*" OR "black american*" OR indian* OR asia* OR aboriginal OR "torres strait islander*" OR "indigenous australian*" OR "first nation") and NOT ("systematic review*" OR "meta-analysis" OR "literature review*")].
Academic Search Ultimate, E-Journals, MedLine, and PsycINFO	Select a Field (TITLE-ABS-KEY): [("intercultural" OR "cultural competenc*" OR "cultural awareness" OR "cultural sensitivity" OR "cultural safety" OR "cultural diversity" OR "cultural humility" OR "cultural literacy") AND ("health care" OR "healthcare" OR "primary care" OR "hospital*" OR "health servic*") AND ("health* professional*" OR "health* provider*" OR "health* work*" OR "health* practitioner*" OR doctor* OR nurs*) AND (program* OR training* OR intervention*) AND (measur* OR evaluation* OR assess* OR outcome*) and NOT ("african american*" OR "black american*" OR indian* OR asia* OR aboriginal OR "torres strait islander*" OR "indigenous australian*" OR "first nation") and NOT ("systematic review*" OR "meta-analysis" OR "literature review*")].
ProQuest- Research Library	Anywhere except full text–NOFT [("intercultural" OR "cultural competenc*" OR "cultural awareness" OR "cultural sensitivity" OR "cultural safety" OR "cultural diversity" OR "cultural humility" OR "cultural literacy") AND ("health care" OR "healthcare" OR "primary care" OR "hospital*" OR "health servic*") AND ("health* professional*" OR "health* provider*" OR "health* work*" OR "health* practitioner*" OR doctor* OR nurs*) AND (program* OR training* OR intervention*) AND (measur* OR evaluation* OR assess* OR outcome*) and NOT ("african american*" OR "black american*" OR indian* OR asia* OR aboriginal OR "torres strait islander*" OR "indigenous australian*" OR "first nation") and NOT ("systematic review*" OR "meta-analysis" OR "literature review*")].
PubPsych<br><br>PubPsych is an online search platform that integrates multiple databases of psycho-logical literature and related fields, including	All these words [("intercultural" OR "cultural competenc*" OR "cultural awareness" OR "cultural sensitivity" OR "cultural safety" OR "cultural diversity" OR "cultural humility" OR "cultural literacy") AND ("health care" OR "healthcare" OR "primary care" OR "hospital*" OR "health servic*") AND ("health* professional*" OR "health* provider*" OR "health* work*" OR "health* practitioner*" OR doctor* OR nurs*) AND (program* OR training* OR intervention*) AND (measur* OR evaluation* OR assess* OR outcome*) and NOT ("african american*" OR "black american*" OR indian* OR asia* OR aboriginal OR "torres strait islander*" OR "indigenous australian*" OR "first nation") and NOT ("systematic review*" OR "meta-analysis" OR "literature review*")]

PSYNDEX-Germany, PASCAL-France, ISOC-Psicología-Spain, MEDLINE®-United States, ERIC-United States, NAR-CIS-Netherlands, NORART-Norway, PsychOpen-Germany, and PsychData-Germany
